# Storage‐Induced Changes in Phenolic Profile and Antioxidant Capacity of Functional Blueberry Beverages Enriched With Propolis

**DOI:** 10.1002/fsn3.72266

**Published:** 2026-08-16

**Authors:** Memnune Sengul, Melek Zor, Seda Ufuk, İsa Arslan Karakutuk, Sefa Aksoy, Elif Feyza Topdas

**Affiliations:** ^1^ Department of Food Engineering, Faculty of Agriculture Atatürk University Erzurum Türkiye; ^2^ Department of Gastronomy and Culinary Arts, School of Tourism and Hotel Management Ağrı İbrahim Çeçen University Ağrı Türkiye

**Keywords:** antioxidant activity, blueberry‐based functional beverages, packaging type, propolis extract, storage temperature, storage time

## Abstract

This study evaluated some physicochemical properties, phenolic profile, and antioxidant activities of blueberry‐based functional beverages fortified with propolis extracts obtained using different solvents (water, glycerol, and propylene glycol). Propolis extract solvent type, storage time (12 months), storage temperature (4°C ± 2°C and 25°C ± 2°C), and packaging type significantly affected the physicochemical characteristics and bioactive properties. Moreover, sample type, storage time, and their interaction significantly affected total phenolic content (TPC), total flavonoid content (TFC), total monomeric anthocyanins (TMA), and antioxidant activities (DPPH, ABTS, and FRAP assays). In line with TPC and TFC, the propolis propylene glycol–water extract enriched blueberry beverage showed the highest antioxidant activity and TMA content. Glass packaging yielded better results in preserving TMA and maintaining the antioxidant activities of DPPH and FRAP than plastic packaging. Caffeic acid, rutin, and resveratrol were detected in all beverages. Principal component analysis showed a clear distinction based on phenolic composition and storage conditions.

## Graphical abstract

Development of functional blueberry beverages enriched with propolis extracts prepared using different solvent systems (water, glycerol–water, and propylene glycol–water). Propolis enrichment and storage conditions influenced the phenolic profile, antioxidant capacity, and physicochemical properties of the beverages during 12 months of storage in glass and PET bottles at 4°C and 25°C.

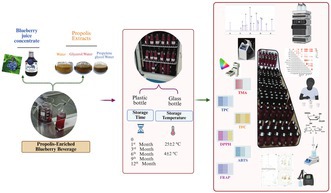

## 1 Introduction

Recently, with increasing consumer awareness of the importance of healthy and balanced nutrition, the demand for fruit‐ and vegetable‐based functional foods has surged (Ashaolu et al. 2025). To meet this demand, the food industry is striving to develop products with low or no additives, aiming to combine practicality, health benefits, and high palatability (Nascimento et al. 2025). In this context, enriching fruit‐based formulations with bioactive extracts to enhance functionality and monitoring the subsequent quality changes during storagerepresents a crucial area of research.

Blueberry ( *Vaccinium corymbosum* L.), a perennial flowering plant in the Ericaceae family, is preferred as a base for fruit beverages due to its rich nutritional content. Widely cultivated and valued worldwide, blueberries are known for their rich nutrients, significant health benefits, delicious flavor, and culinary versatility (Karami et al. 2025). Blueberries contain a variety of bioactive substances, including flavonoids, anthocyanins, fatty acids, polyphenols, and lignans, along with important nutrients like fiber, amino acids, minerals, and vitamins (Arshad et al. 2025; Kim, Cho, and Lee 2025; Kim, Kwon, et al. 2025). These bioactive compounds have been associated with various protective health benefits, including reduced risks of cardiovascular disease, cancer, diabetes, and oxidative stress‐related conditions (Falcone et al. 2025; Kim, Cho, and Lee 2025; Kim, Kwon, et al. 2025; Onuh et al. 2023). They have become a common part of diets worldwide, as they are widely consumed fresh, frozen, or processed into juices, jams, and baked goods (Arshad et al. 2025; Tobar‐Bolaños et al. 2021).

Propolis is a natural, heterogeneous substance produced by honeybees (*Apis mellifera L*.) by mixing botanically sourced resins and saps with beeswax and salivary enzymes, making its extracts highly valuable for functional foods (Balasubramaniam et al. 2025; Martinotti et al. 2025). Its complex chemical composition varies significantly based on the queen bee's genetics, geographical location, climate, season, botanical sources, and extraction solvents (Lazo et al. 2025; Wozniak et al. 2019; Yavas and Bastürk 2024). It primarily consists of a diverse range of organic compounds, such as flavonoids, terpenes, and phenolic acids, alongside wax, essential oils, pollen, and trace elements (Can et al. 2024; Kurek‐Górecka et al. 2025). Due to this rich bioactive profile, propolis serves as a natural preservative and health enhancer in the food industry, exhibiting potent antioxidant (Ufuk and Sengul 2023), antimicrobial (Lazo et al. 2025), anti‐inflammatory (Kowalczyk et al. 2025), antiviral (Ferreira et al. 2025), and anticancer (Klósek et al. 2025) activities.

Because raw propolis has an intense flavor and is unsuitable for direct human consumption, extraction is necessary to utilize its components (Hangişi 2024). Although its sensitivity to processing conditions poses application challenges, propolis holds significant economic potential, and its market is expected to grow across various food sectors (Ballouk et al. 2025). However, while various methods and solvents are employed for extraction (Rompas et al. 2025), research regarding the direct application of these extracts in food products remains limited.

Like most liquid formulations, polyphenol‐rich functional beverages are highly susceptible to changes in physicochemical characteristics, phenolic composition, and antioxidant activity during storage (Alim et al. 2023; Zhang et al. 2025). Therefore, packaging material and storage temperature play important roles in maintaining the physicochemical quality, phenolic composition, and antioxidant properties of beverages throughout storage (Edo et al. 2025; Fadiji et al. 2023).

While various functional beverages have been reported in the literature (Cilla et al. 2019; Vilas‐Boas et al. 2022), research on a blueberry‐based beverage enriched with different propolis extracts and its storage stability is lacking. Building upon a previous study that characterized propolis extracts obtained via water, glycerol–water, and propylene glycol–water systems at the extract level, finding that propylene glycol–water extracts offered superior phenolic and antioxidant performance (Sengul et al. 2026), this study evaluates how these solvent‐dependent characteristics behave within a real food matrix. Consequently, this study reports the formulation of a blueberry‐based beverage fortified with these solvent‐specific propolis extracts and investigates its stability over a 12‐month storage period using different packaging materials (glass and PET) and temperatures. In conclusion, the aim of this study was to evaluate the influence of propolis extracts prepared with different solvents on the phenolic profile and antioxidant properties of blueberry beverages and to determine the effects of storage duration, temperature, and packaging type on these properties during storage.

## 2 Materials and Methods

### 2.1 Materials

In this study, raw propolis was obtained from the Artvin Beekeepers Association in August 2023. The source of the propolis is Aydın village in the Ardanuç district of Artvin, Türkiye (41°02′37″ N, 42°05′44″ E). After acquisition, the propolis and blueberry concentrate were stored at −20°C until used for production to preserve their properties. The 100% blueberry concentrate used in the fruit beverages was sourced from Brownwood Acres Foods Inc., Canada. The glass and plastic (PET) bottles used for packaging were supplied by a company in Bursa, Türkiye.

### 2.2 Methods

In this study, a blueberry beverage was prepared from blueberry concentrate and enriched with 6% propolis extracts obtained using different solvents. The beverages were stored in glass (control) and plastic packaging at room temperature (25°C ± 2°C) and refrigerator temperature (4°C ± 2°C) in the dark for 12 months. Analyses of the beverages were conducted during the storage period (0, 1, 3, 6, 9, 12 months).

#### 2.2.1 Preparation of Extracts From Raw Propolis

The propolis extracts used in the beverage formulation were prepared with slight modifications to the procedure described by Topdas et al. (2020), as explained in our previous study (Sengul et al. 2026). For this purpose, crude propolis was ground and sieved by a stainless steel sieve (0.45 mm), and 25 g of powder was extracted with 250 mL of pure water, propylene glycol–water (1:1, v/v), or glycerol–water (1:1, v/v). Extraction was performed at 35°C with agitation at 60 rpm for 24 h in a shaking water bath (JSR, JSSB‐30 T, Korea). Extracts were then filtered, centrifuged, and the supernatants collected. The procedure was done in triplicate, and extracts were stored at −20°C until beverage production.

#### 2.2.2 Production of Blueberry Beverage

**FIGURE 1:**
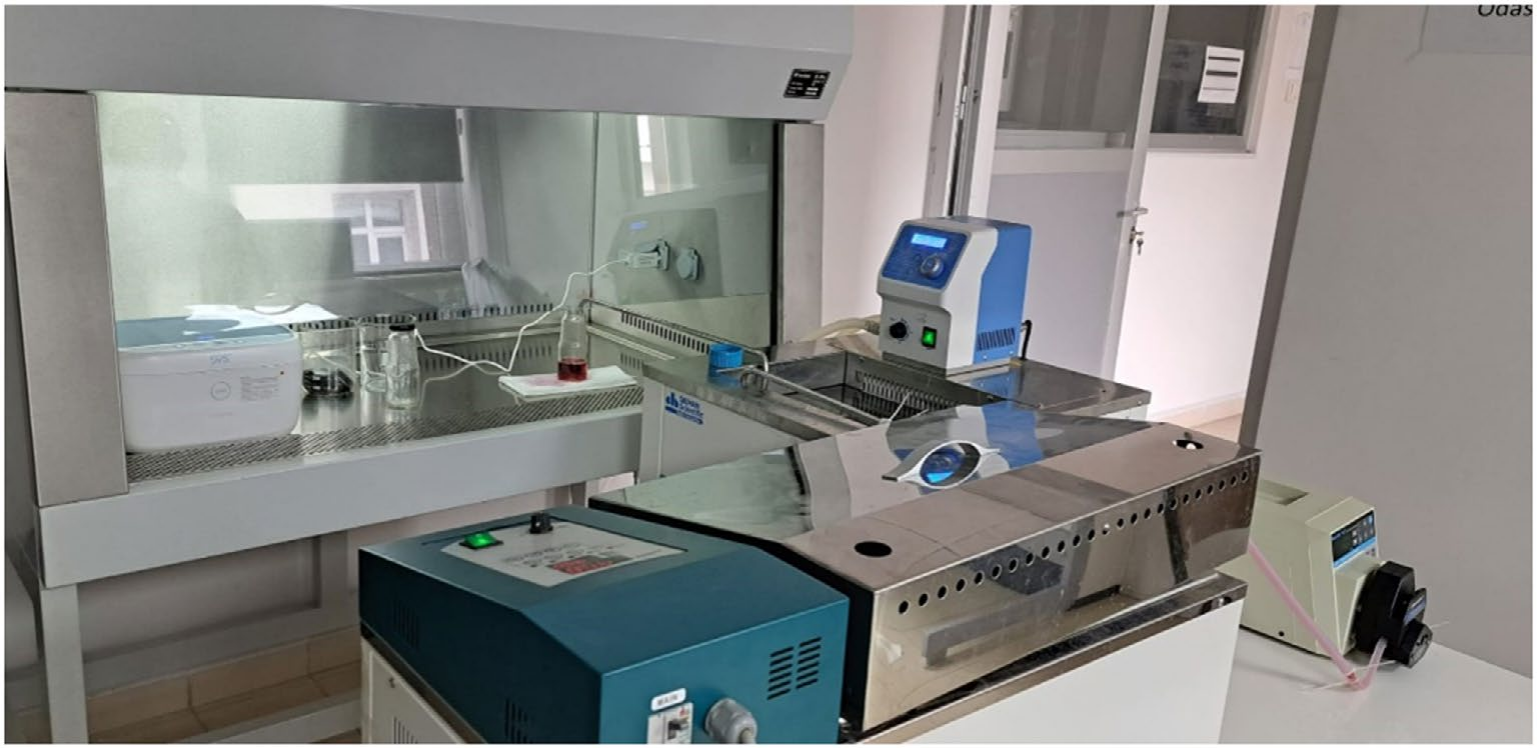
Blueberry beverage preparation apparatus.

Blueberry ( *Vaccinium corymbosum* L.) beverages were prepared from blueberry concentrate using the formulation and production procedure adapted from Ufuk (2023), who developed the original formulation using bilberry ( *Vaccinium myrtillus* L.). The 6% propolis extract concentration used in this study was determined from preliminary formulation studies conducted before the trial. Different propolis concentrations were evaluated, and it was observed that at higher concentrations, the intense taste and aroma characteristic of propolis negatively affected product acceptance, while at lower concentrations, the targeted functional contribution remained limited. Therefore, the 6% concentration was preferred because it provided a suitable balance between functional properties and sensory acceptability. Glass bottles (200 mL) for the fruit beverages were sterilized in an autoclave, while plastic bottles were sterilized with UV light. The blueberry beverages (controls) were filled into sterile glass and plastic bottles after pasteurization (at 90°C for 1 min) (Yang et al. 2017). The setup for preparing the fruit beverages is shown in Figure 1. Four beverage formulations were included in the study: a control blueberry beverage without propolis addition (C), a blueberry beverage enriched with propolis water extract (PW‐BB), a blueberry beverage enriched with propolis glycerol–water extract (PGly‐BB), and a blueberry beverage enriched with propolis propylene glycol‐water extract (PPG‐BB). All beverage formulations were packaged in both glass and plastic bottles and stored under the specified storage conditions.

#### 2.2.3 Color Intensity

The color intensity of fruit beverages was determined using a Konica Minolta Colorimeter (Konica Minolta CR‐400, Korea). Color measurements were performed according to CIELAB (L*, a*, b*) color space parameters. The color parameters of blueberry beverages were measured using clear glass cuvettes with an optical path length of 10 mm. Measurements were taken under standard illumination and a standard viewing angle of 10°. The device was calibrated before use. Color intensity values (L* (lightness‐darkness), a* (red‐green), b* (yellow‐blue), Chroma (C*), and Hue (H°)) were determined using the CIELAB color system (Karakütük et al. 2023).

#### 2.2.4 Determination of Soluble Solid Content (SSC)

The SSC of fruit beverages was determined using a digital refractometer (WYA 2S, Shanghai Physico‐Optical Instrument Factory, China) (Cemeroğlu 2013).

#### 2.2.5 pH and Titratable Acidity (TA) Analyses

The pH values of the samples were first measured using a pH meter (OHAUS Starter 3100, USA model) calibrated with pH 4.00, 7.00, and 10.00 buffers at room temperature. TA was measured by titrating 30 mL of fruit beverages with added propolis extract with 0.1 M NaOH until the pH was 8.1 using a pH meter (OHAUS Starter 3100, USA model). Titration acidity was calculated as g/100 mL of anhydrous citric acid (CA) (Yang et al. 2017).

#### 2.2.6 Determination of Sugar, Sucrose, and Reducing Sugar Contents

The sugar content of fruit beverages was determined using the Lane‐Eynon method. The amount of naturally occurring reducing sugars was calculated using the value determined before the inversion, and the amount of sucrose was calculated by multiplying the difference between the values determined before and after the inversion by 0.95. The total sugar content was determined by adding the amount of naturally occurring reducing sugars and sucrose (Cemeroğlu 2013).

#### 2.2.7 Determination of Total Monomeric Anthocyanins (TMA)

Fruit beverages were individually diluted with KCl (Potassium Chloride) buffer at pH 1.0 and with C_2_H_3_NaO_2_ (Sodium Acetate) buffer at pH 4.5. Absorbance was measured at 700 nm and 515 nm using a visible spectrophotometer (PG Instruments T60V, UK). Values are calculated using Equation (1). Measured values are expressed in mg cyanidin‐3‐glucoside (C3G) equivalents per 100 mL (Cemeroğlu 2013).

(1) $$\mathrm{TMA}\;\left(\mathrm{mg}\;C3G/100\;\mathrm{mL}\right)=\left(A\times \mathrm{MW}\times \mathrm{Df}\times 100\right)/\varepsilon \times l$$

In Equation (1), A represents the absorbance value (*A* = (*A*_515_−*A*_700_) pH_1.0_−(*A*_515_−*A*_700_) pH_4.5_). The anthocyanin content was calculated as C3G equivalents (Molecular weight (MW) = 449.20 g/mol; *ε* = 26,900 L/(mol × cm)). Spectrophotometric measurements were performed at 515 nm and 700 nm using cuvettes with a 1 cm path length (ℓ), with the dilution factor (Df) taken into account.

#### 2.2.8 Extraction of Samples

For extraction, 20 mL of the fruit beverage was taken, 20 mL of 100% methanol was added, and the mixture was extracted in an ultrasonic water bath for 30 min at a temperature not exceeding 35°C. The resulting extract was centrifuged at 4°C at 6000 rpm for 15 min. Then, the supernatant was filtered through Whatman No. 1 filter paper, and the extracts were stored at −20°C until analysis (Karakütük et al. 2023). The prepared extracts were used to determine total phenolic content (TPC), total flavonoid content (TFC), and antioxidant activity analyses (DPPH (1,1‐diphenyl‐2‐picrylhydrazyl), ABTS (2,2′‐azino‐bis(3‐ethylbenzthiazolin‐6‐sulfonic acid), and FRAP (ferric ion reducing power))).

#### 2.2.9 Determination of TPC and TFC

TPC analysis was performed by making minor modifications to the method reported by Singleton et al. (1999). First, 2.5 mL of 0.2 N Folin–Ciocalteu reagent (FCR) was pipetted into 0.1 mL of the extract and vortexed. After 3 min of incubation, 2 mL of 7.5% Na_2_CO_3_ solution was added, the mixture was vortexed, and incubated in the dark at room temperature for 2 h. After incubation, absorbance was measured at 760 nm using a visible spectrophotometer. The TPC of the beverages was calculated using an equation and expressed as mg gallic acid equivalents per 100 mL (mg GAE/100 mL) using an equation derived from a graph prepared with gallic acid standards at concentrations of 10–500 μg/mL. The analysis was performed three times for each sample.

The TFC of the beverages was analyzed according to the protocol reported by Koçak et al. (2018). A 0.25 mL sample was taken, and 1.25 mL of pure water and 0.075 mL of 0.05 g/mL NaNO_2_ were added, then vortexed. After waiting 6 min, 0.15 mL of 0.1 g/mL AlCl_3_·6H_2_O was added, vortexed, and allowed to stand for another 5 min. 0.5 mL of 1 M NaOH was added, vortexed, and allowed to stand for 15 min. Sample absorbances were recorded at 510 nm using a visible spectrophotometer. TFC was calculated from the calibration curve using quercetin standards (10–750 mg/L) and expressed as mg quercetin equivalents per 100 mL (mg QE/100 mL). The analysis was performed in triplicate.

#### 2.2.10 Determination of Antioxidant Activity by DPPH Method

DPPH (1,1‐diphenyl‐2‐picrylhydrazyl) free radical scavenging activity is analyzed using the method reported by Balaydin et al. (2010). For this purpose, 25 μL of each extract was pipetted into a test tube, and then the volume was completed to 2.5 mL with the prepared DPPH stock solution (0.1 mM). The vortexed tubes were incubated at room temperature in the dark for 30 min. Absorbances were measured at 517 nm. The analysis was performed three times for each sample. % Inhibition values were calculated using Equation (2) (Sengul et al. 2023). A calibration curve was created by determining the inhibition % values for different Trolox concentrations (5–25 μM), and the antioxidant capacity of each sample was reported as μM Trolox Equivalent (TE)/100 mL sample from this calibration curve (Cemeroğlu 2007; Zor et al. 2024).

(2) $$\text{Inhibition}\%=\left[\left(A\text{DPPH or ABTS}-A\text{Sample}\right)/A\text{DPPH or ABTS}\right]\times 100$$

#### 2.2.11 Determination of Antioxidant Activity by ABTS Method

The procedure reported by Köksal et al. (2009) was used to determine the free radical scavenging activity of ABTS (2,2′‐azino‐bis(3‐ethylbenzthiazoline‐6‐sulfonic acid)). The ABTS+ radical solution was prepared by adding 2.45 mM potassium persulfate solution to 7 mM ABTS solution and incubating for 16 h at ambient temperature in the dark. 25 μL of each extract was added to the test tubes. The total volume was brought to 2.5 mL using the prepared ABTS• + radical solution. The tubes were then vortexed and kept in the dark for 6 min. Absorbances were measured at 734 nm using a visible spectrophotometer. The analysis was performed three times. Inhibition % values were calculated using Equation (2) (Sengul et al. 2022; Topdas et al. 2020). A calibration curve was obtained for different concentrations of Trolox (5–25 μM). Antioxidant capacity was determined as % inhibition values and expressed as μM TE/100 mL sample according to this curve (Cemeroğlu 2007; Zor et al. 2024).

Here, *A*_DPPH_ or *A*_ABTS_ represents the absorbance of the DPPH and ABTS blank sample (depending on the analysis performed), and *A*_sample_ represents the absorbance value of the beverage.

#### 2.2.12 Determination of Antioxidant Activity by Ferric Reducing Antioxidant Power (FRAP) Assay

The antioxidant capacity of beverage samples was evaluated using the FRAP assay with slight modifications to the method described by Koçak et al. (2018). This assay relies on the ability of antioxidants to reduce Fe^3+^ ions in the (Fe(TPTZ)^3+^) complex to the blue‐colored (Fe(TPTZ)^2+^) complex under acidic conditions. Three distinct solutions were prepared prior to analysis: an acetate buffer (pH 3.6) composed of 3.1 g sodium acetate and 16 mL acetic acid, a TPTZ solution containing 0.156 g of 2,4,6‐tripydryl‐s‐triazine (TPTZ) dissolved in 50 mL of ethanol, and a ferric chloride solution prepared with 0.5404 g of FeCl_3_·6H_2_O and 2 mL of 37% (m/m) HCl.

The FRAP reagent was prepared by mixing 10 mL of solution 1, 1 mL of solution 2, and 1 mL of solution 3 in a volumetric flask. For each measurement, 10 μL of the sample was added to 990 μL of the FRAP reagent, mixed thoroughly, and allowed to react for 4 min. Absorbance readings were recorded at 593 nm using a spectrophotometer. The results were expressed as μM of Trolox Equivalents (μM TE) per 100 mL of sample.

#### 2.2.13 Phenolic Compound Profile by HPLC

Solid‐phase extraction (SPE) was applied to purify phenolic compounds. Before use, SPE cartridges were conditioned correctly. For this purpose, the cartridges were activated with 5 mL of methanol followed by 5 mL of acidified water (0.01%). After conditioning, 1 mL of the sample was loaded into the cartridge to interact with the cartridge matrix. Following sample loading, the cartridge was washed with 1 mL of acidified water to remove matrix interferences. Then, 5 mL of methanol was passed through the cartridge to elute the target compounds. The resulting methanolic eluate was evaporated on a rotary evaporator at 35°C until the methanol was completely removed. After drying, the residue was re‐dissolved in 1 mL of methanol. In the final step, the solution was filtered through a 0.45 μm PTFE syringe filter to remove particles and stored in vials until analysis (Coklar and Akbulut 2017).

The phenolic component profile of the extracts was analyzed using an Agilent 1260 Infinity Series HPLC (High Performance Liquid Chromatography) system equipped with a 1260 quantitative pump, a 1260 Infinity standard autosampler, and a 1260 VWD UV detector (set to 280 nm), with modifications to the method reported by Kara et al. (2022). A reverse‐phase C18 column (100 Å, 5 μm, 250 × 4.6 mm inner diameter) was used for chromatographic separation. The column temperature was 30°C. The mobile phases were ultrapure water containing 0.03% phosphoric acid (pH 2.5) (A) and acetonitrile (B). The mobile phase flow rate and injection volume were 0.6 mL/min and 5 μL, respectively. Total analysis and post‐run times were 43 min and 2 min, respectively. The gradient program was initiated by maintaining mobile phase A at 95% for the first 8 min, then gradually decreasing to 85%, 79%, 65%, 60%, 48%, and 33% at min 8, 10, 15, 17, 20, and 36, respectively. From this point, mobile phase A was increased back to 95% at min 36.1 and held at this level until min 43. Between min 43 and 45, the system transitioned to the post‐run phase. Standard phenolic compounds (gallic acid, epigallocatechin, chlorogenic acid, catechin, epicatechin, caffeic acid, rutin, *p*‐coumaric acid, trans‐sinapic acid, ferulic acid, myricetin, resveratrol, luteolin, quercetin, apigenin, diosmetin, sinensetin, and chrysanthemum) were prepared for analysis by dissolving them in a mixture of 80% methanol and 20% pure water (v/v). Identification of phenolic compounds was confirmed by comparing retention times and UV spectra with reference standards, and data were analyzed using ChemStation software. All samples were filtered through 0.45 μm membrane filters before being placed in the instrument.

For each phenolic compound, calibration curves were generated using solutions prepared by serially diluting standard solutions in the range of 1–50 ppm. Calibration curves were plotted as a function of the obtained concentration (*x*‐axis) versus the peak area (*y*‐axis). To determine the sensitivity of the method, limit of detection (Limit of detection (LOD), 3.3 × SD/m) and limit of quantification (Limit of quantification (LOQ), 10 × SD/m) values were calculated. These limit values were determined based on the standard deviation (SD) of the measurement data obtained at the lowest standard concentration and the slope (m) of the calibration curve. The RP‐HPLC‐UV analysis parameters of the beverages are given in the Table S1.

#### 2.2.14 Sensory Analysis

Sensory evaluation was conducted on freshly prepared blueberry beverages. The beverages were evaluated in terms of appearance, color, odor, flavor, mouthfeel, and overall acceptability. Sensory evaluation was conducted by 20 semi‐trained panelists consisting of faculty members and graduate students from the Department of Food Engineering, Faculty of Agriculture, Atatürk University. All procedures were approved by the Atatürk University Ethics Committee (Report no: E‐38813508‐010.09‐2,600,198,186) and conducted in accordance with the Declaration of Helsinki. The samples were served at room temperature in identical containers coded with random three‐digit numbers and presented in a randomized order. A five‐point scoring scale was used, where 1 = poor, 2 = average, 3 = good, 4 = very good, and 5 = excellent. Water was provided to the panelists for palate cleansing between samples. Individuals with known allergies to bee products were excluded from the sensory evaluation. All participants were informed about the evaluation procedure and provided voluntary consent before participating.

#### 2.2.15 Statistical Analyses

The study was conducted in a tri‐parallel configuration according to a fully randomized experimental design with a factorial structure of 3 beverages × 2 packaging types (glass and plastic) × 2 storage temperatures (4°C and 25°C) × 6 storage durations (0, 1, 3, 6, 9, and 12 months). Blueberry beverages were prepared and packaged using standard production procedures. Samples were stored at two temperatures and analyzed at specified storage times. All analyses were performed as three parallel measurements. The results obtained were statistically evaluated using multivariate analysis of variance (ANOVA) in SPSS version 25.0 (IBM, Armonk, New York, USA). Duncan's multiple‐comparison test was used to determine significant differences among factors and their interactions. All findings are presented as mean ± standard deviation, and statistical significance was set at *p* < 0.01. To examine the structural pattern of the phenolic component data and the relationships between variables in more detail, Principal Component Analysis (PCA) was performed using SIMCA 14.1 software (MKS Umetrics, Umea, Sweden).

## 3 Results and Discussion

### 3.1 Physicochemical Properties

The color parameters, pH, titratable acidity, SSC, reducing sugar, sucrose, and total sugar values of blueberry beverages with different types of propolis extracts at different types of packaging and storage conditions for 12 months are given in Table 1. The type of solvent used for propolis extraction had a statistically significant effect on color, pH, titratable acidity, SSC, and sugar composition (*p* < 0.01). Among the sample types, the control group (C) had the highest lightness (L*), while beverages with propylene glycol extract (PPG‐BB) had the lowest. According to a study conducted by Albaş et al. (2022), the addition of lactic‐acid–based propolis to fresh strawberry juice decreased the L* value of the samples compared to the control group. During storage, L* value increased, indicating lightening over time. Among the sample types, PPG‐BB has the lowest a* value, while the beverage with glycerol extract (PGly‐BB) has the highest a* value. Over time, the a* value decreases, indicating a shift from red to green during storage. Compared to the C group, the pH values of the beverages increased with the addition of propolis. According to Ufuk (2023), the pH value of the C group was lower than that of other beverages. The highest SSC was observed in the PGly‐BB. This indicates that glycerol has a high solubilizing capacity and can increase SSC. With the addition of propolis extracts, the Brix values were higher compared to the C group. According to a study by Bağdat and Ilıkkan (2024), adding propolis extracts to kefir beverages increased °Brix levels compared with the control group. This increase was attributed to the soluble solids present in propolis. PGly‐BB and PPG‐BB have slightly lower reducing sugar content. This may be due to interactions between glycerol and propylene glycol and reducing sugars. The addition of propolis extracts, especially PPG‐BB and PGly‐BB, may increase the sucrose value in the beverages compared with the C group.

**TABLE 1:** Some physicochemical properties of blueberry beverages.

Sample type	L*	a*	b*	C*	H°	pH	Titratable acidity	Soluble solids content	Reducing sugars	Sucrose	Total sugar
C	48.43 ± 3.33^a^	12.33 ± 6.61^c^	5.17 ± 1.34^c^	13.90 ± 5.55^b^	28.82 ± 18.46^b^	2.71 ± 0.08^c^	0.32 ± 0.06^d^	12.58 ± 0.38^d^	5.88 ± 4.16^a^	5.69 ± 3.93^d^	11.57 ± 0.35^c^
PW‐BB	47.02 ± 2.66^b^	13.57 ± 5.87^b^	6.54 ± 1.65^a^	15.55 ± 4.68^a^	29.82 ± 15.87^a^	2.76 ± 0.10^a^	0.32 ± 0.05^c^	12.62 ± 0.35^c^	5.44 ± 3.89^b^	6.15 ± 3.61^c^	11.59 ± 0.36^b^
PGly‐BB	46.34 ± 2.71^c^	14.02 ± 5.69^a^	6.12 ± 1.88^b^	15.82 ± 4.43^a^	27.57 ± 16.26^c^	2.74 ± 0.09^b^	0.33 ± 0.05^b^	15.33 ± 0.33^a^	5.25 ± 3.91^d^	6.23 ± 3.63^b^	11.48 ± 0.36^d^
PPG‐BB	35.44 ± 2.43^d^	9.83 ± 2.46^d^	5.12 ± 2.90^c^	11.41 ± 2.57^c^	27.93 ± 13.18^c^	2.74 ± 0.09^b^	0.34 ± 0.05^a^	14.65 ± 0.31^b^	5.35 ± 3.92^c^	6.27 ± 3.71^a^	11.62 ± 0.27^a^
Significance level	**	**	**	**	**	**	**	**	**	**	**
**Storage time**											
0	41.10 ± 4.27^e^	19.32 ± 3.41^a^	3.82 ± 0.43^d^	19.67 ± 3.45^a^	11.49 ± 1.87^f^	2.84 ± 0.03^b^	0.42 ± 0.01^a^	13.90 ± 0.98^c^	1.14 ± 0.06^f^	10.18 ± 0.14^a^	11.32 ± 0.10^c^
1st month	43.82 ± 4.89^c^	15.12 ± 2.86^c^	4.85 ± 0.84^c^	15.92 ± 2.76^c^	18.26 ± 4.11^e^	2.85 ± 0.07^a^	0.37 ± 0.02^b^	14.12 ± 1.32^a^	3.06 ± 1.71^e^	8.27 ± 1.70^b^	11.32 ± 0.10^c^
3rd month	42.00 ± 6.34^d^	15.28 ± 5.14^b^	6.47 ± 3.38^a^	16.87 ± 5.33^b^	23.03 ± 9.78^d^	2.67 ± 0.03^e^	0.30 ± 0.03^c^	14.06 ± 1.25^b^	5.22 ± 3.17^d^	6.11 ± 3.15^c^	11.33 ± 0.11^c^
6th month	46.72 ± 4.95^a^	9.84 ± 3.31^d^	6.60 ± 1.49^a^	12.15 ± 2.40^d^	35.75 ± 13.02^c^	2.69 ± 0.02^d^	0.28 ± 0.01^f^	13.60 ± 1.31^d^	7.25 ± 3.70^c^	4.63 ± 3.66^d^	11.88 ± 0.16^a^
9th month	45.46 ± 5.96^b^	7.98 ± 2.91^e^	5.89 ± 1.44^b^	10.22 ± 2.05^e^	38.23 ± 14.16^b^	2.64 ± 0.03^f^	0.29 ± 0.01^e^	13.61 ± 1.30^d^	7.85 ± 3.56^b^	3.79 ± 3.23^e^	11.65 ± 0.35^b^
12th month	46.74 ± 6.32^a^	7.08 ± 2.48^f^	6.81 ± 1.87^a^	10.18 ± 1.53^e^	44.45 ± 15.25^a^	2.73 ± 0.05^c^	0.30 ± 0.02^d^	13.47 ± 1.33^e^	8.35 ± 3.69^a^	3.53 ± 3.35^f^	11.88 ± 0.36^a^
Significance level	**	**	**	**	**	**	**	**	**	**	**
**Storage temperature**											
25°C ± 2°C	45.16 ± 5.99^a^	10.66 ± 5.57^b^	6.51 ± 2.46^a^	13.14 ± 4.50^b^	35.78 ± 18.06^a^	2.76 ± 0.10^a^	0.32 ± 0.06^b^	13.91 ± 1.26^a^	7.53 ± 4.58^a^	4.15 ± 4.27^b^	11.68 ± 0.39^a^
4°C ± 2°C	43.45 ± 5.68^b^	14.22 ± 5.08^a^	4.96 ± 1.29^b^	15.20 ± 4.79^a^	21.29 ± 9.02^b^	2.71 ± 0.08^b^	0.33 ± 0.05^a^	13.68 ± 1.27^b^	3.43 ± 1.40^b^	8.02 ± 1.36^a^	11.44 ± 0.22^b^
Significance level	**	**	**	**	**	**	**	**	**	**	**
**Packaging type**											
Plastic	44.93 ± 6.16^a^	11.57 ± 5.98^b^	6.04 ± 2.48^a^	13.68 ± 4.97^b^	32.16 ± 18.33^a^	2.73 ± 0.10^b^	0.33 ± 0.05^a^	13.84 ± 1.28^a^	5.51 ± 4.03^b^	6.06 ± 3.77^b^	11.57 ± 0.34
Glass	43.64 ± 5.55^b^	13.35 ± 5.08^a^	5.45 ± 1.62^b^	14.70 ± 4.47^a^	24.86 ± 12.34^b^	2.74 ± 0.09^a^	0.32 ± 0.05^b^	13.76 ± 1.26^b^	5.45 ± 3.91^a^	6.11 ± 3.67^a^	11.56 ± 0.34
Significance level	**	**	**	**	**	**	**	**	**	**	NS
S × STi	**	**	**	**	**	**	**	**	**	**	**
S × STe	**	**	NS	**	**	**	**	**	**	**	NS
S × *P*	**	**	NS	**	**	**	**	**	**	**	NS
STi × STe	**	**	**	**	**	**	**	**	**	**	**
STi × *P*	**	**	NS	**	**	**	**	**	**	**	**
STe × *P*	*	**	*	**	**	NS	**	*	**	**	NS

*Note:* Different superscript letters (a–e) within the same column indicate significant differences (*p* < 0.01). Units are expressed as g/100 mL for TA (CA, citric acid), reducing sugars, sucrose, and total sugars, and as °Brix for soluble solids content (SSC).

Abbreviations: C, Control group; NS, Not Significant; P, Packaging Type; PGly‐BB, Propolis glycerol–water extract enriched blueberry beverage; PPG‐BB, Propolis propylene glycol–water extract enriched blueberry beverage; PW‐BB, Propolis water extract enriched blueberry beverage; S, Sample Type; STe, Storage Temperature; STi, Storage Time.

The storage temperature (room and refrigerator temperature) and package type (glass and plastic packaging) had statistically significant effects on color, pH, titratable acidity, SSC, and sugar composition (*p* < 0.01), as shown in Table 1. Storage at room temperature and in plastic packaging increased the L*, b*, and C* parameters. Glass packaging preserved pH better than plastic packaging, likely due to lower oxygen and light permeability. Moreover, SSC is calculated to be higher in samples stored in plastic bottles. This may be due to the higher oxygen permeability of the plastic. Similar storage‐dependent changes in soluble solids content have also been reported in fruit‐based beverages (Jacob et al. 2023). According to the results, reducing sugar content increases more at room temperature. Under refrigerated conditions, chemical and hydrolysis reactions slow down leading to lower reducing sugar content. Moreover, plastic packaging has a higher content of reducing sugars. This may be due to the higher oxygen permeability of plastic than glass. In addition, the sucrose level is higher under refrigeration conditions. Refrigerated storage slows the hydrolysis of sucrose and the degradation of sugars. In glass packaging, the sucrose level is higher than in plastic packaging due to glass's inert properties. Glass packaging provides greater stability for beverages, reduces oxygen exposure, and may slow sucrose degradation. At room temperature, the total sugar levels are higher than at refrigerator temperature.

The storage time (0, 1st, 3rd, 6th, 9th, 12th months) had a statistically significant effect on color, pH, titratable acidity, SSC, and sugar composition (*p* < 0.01), as shown in Table 1. Over time, the H° value increases at room temperature and in the plastic packaging. Similar changes in color parameters during storage have been reported previously for fruit beverages stored under different packaging conditions (Pérez‐Vicente et al. 2004). Comparable storage‐related changes in color attributes and anthocyanin degradation have also been reported in sour cherry juices (Wojdylo et al. 2019). Over time, TA decreases, likely due to chemical reactions or enzymatic degradation. Mgaya‐Kilima et al. (2015) evaluated roselle–mango juice blends (20%, 40%, 60%, and 80% extract) stored in glass and plastic bottles at 4°C and 28°C for up to 6 months. Throughout storage, titratable acidity (TA) gradually declined in both bottle types due to the metabolic consumption and degradation of organic acids. Soluble solids content (SSC) slightly increased during the first 3 months, driven by the dissolution and migration of extract components, before decreasing due to sedimentation and component degradation. Furthermore, sucrose levels continuously decreased as it hydrolyzed into reducing sugars, resulting in a corresponding, significant increase in total reducing sugars over time. Similar increases in reducing sugars accompanied by decreases in sucrose during storage have been reported previously and are generally attributed to sucrose hydrolysis under acidic conditions (Akhtar et al. 2013).

### 3.2 Sensory Properties

**FIGURE 2:**
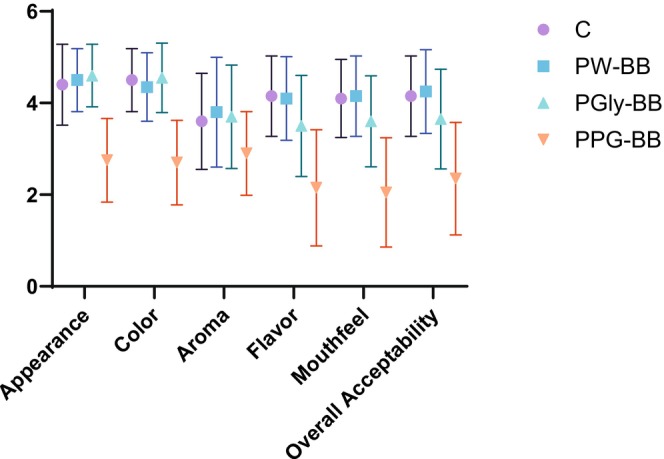
Sensory evaluation results for blueberry beverages. C, Control group; PGly‐BB, Propolis glycerol–water extract enriched blueberry beverage; PPG‐BB, Propolis propylene glycol–water extract enriched blueberry beverage; PW‐BB, Propolis water extract enriched blueberry beverage.

The sensory evaluation of the blueberry beverages was conducted by panelists based on parameters such as appearance, color, mouthfeel, flavor, aroma, and overall acceptability. The sensory test results for the blueberry beverages, evaluated by the panelists, are presented in Figure 2.

Regarding visual quality, the PGly‐BB recorded the highest appearance score (4.60 ± 0.68) and retained its leadership in the color parameter (4.55 ± 0.76). On the contrary, the PPG‐BB was the least appreciated by the panelists for both appearance (2.75 ± 0.91) and color (2.70 ± 0.92). These results suggest that the use of PGly may have improved the stability and visual vibrancy of blueberry pigments, while the PPG formulation may have contributed to lower visual acceptance by the panelists.

When the odor and taste profiles of the beverages were examined, the highest aroma score was observed for PW‐BB (3.80 ± 1.20), while the lowest score (2.90 ± 0.91) was again recorded for PPG‐BB. In terms of flavor, the C group received the highest result (4.15 ± 0.88), which may reflect the panelists' familiarity with the traditional taste of blueberry. In the mouthfeel category, which directly affects the panelists' experience, PW‐BB emerged as the most successful formulation with a 4.15 ± 0.88 score, while PP‐BB showed a noteworthy weakness in this category (2.05 ± 1.19). This low score may indicate that the panelists perceived the formulation less favorably in terms of mouthfeel.

The overall acceptability results, which synthesize all sensory parameters, indicate that PW‐BB is the most preferred formulation among panelists, with a score of 4.25 ± 0.91. In contrast, PPG‐BB was classified as the least preferred beverage, consistently receiving the lowest scores across all data, including an overall acceptability of 2.35 ± 1.23. These data indicate that the ingredients used in blueberry beverage formulations play a pivotal role not only in taste but also in the beverage's visual and textural identity. In addition, the results highlight that the integrating aroma and mouthfeel is crucial for the success of the final product in development processes.

### 3.3 TPC, TFC, TMA, and Antioxidant Activity

Table 2 presents the effects of various factors (sample type, storage time, storage temperature, and packaging) on the TPC, TFC, TMA, and antioxidant activity results of blueberry beverages. The comprehensive evaluation showed that sample type and storage time had statistically significant effects on the stability of phenolics, flavonoids, anthocyanins, and other antioxidant compounds, compared to interactions among other variables. The TPC and TFC contents of the beverages (PW‐BB, PGly‐BB, and PPG‐BB) ranged from 48.09 to 77.42 mg GAE/100 mL and 38.92 to 69.56 mg QE/100 mL, respectively. Similar to TPC and TFC results, the PPG‐BB group showed the highest antioxidant activity (DPPH, ABTS, and FRAP) and TMA content, with values of 195.81 μM TE/100 mL, 212.77 μM TE/100 mL, 296.83 μM TE/100 mL, and 1.31 mg C3G/100 mL, respectively. This result indicates that beverages containing propylene glycol–water extract exhibited higher levels of phenolic compounds and antioxidant activity than the other beverage groups. It aligns with previous findings (Sengul et al. 2026) showing that propylene glycol–water extracts had the highest total phenolic content, flavonoid concentration, and antioxidant capacity among tested solvents. Myo et al. (2025) similarly reported higher extraction yields of several polar and semi‐polar phytochemicals when propylene glycol–water solvent systems were used. In all analyses except the TMA, the PPG‐BB group was followed by the PGly‐BB, PW‐BB, and C groups, respectively. Consistent with our findings, many studies have shown that polyol‐water solvents perform better than water (Myo and Khat‐udomkiri 2022; Win et al. 2024).

**TABLE 2:** Total phenolic, total flavonoid, total monomeric anthocyanin, and antioxidant assays of the blueberry beverages.

Sample Type	TPC	TFC	DPPH	ABTS	FRAP	TMA
C	37.66 ± 5.47^d^	30.50 ± 6.02^d^	123.01 ± 28.82^d^	128.42 ± 19.10^d^	175.13 ± 45.09^d^	1.16 ± 1.09^b^
PW‐BB	48.09 ± 4.90^c^	38.92 ± 6.06^c^	154.31 ± 42.10^c^	179.01 ± 53.36^c^	214.13 ± 38.77^c^	1.16 ± 1.05^b^
PGly‐BB	53.67 ± 5.13^b^	43.70 ± 6.66^b^	167.07 ± 38.43^b^	181.29 ± 40.13^b^	228.15 ± 27.49^b^	1.16 ± 0.98^b^
PPG‐BB	77.42 ± 6.10^a^	69.56 ± 7.92^a^	195.81 ± 41.57^a^	212.77 ± 22.89^a^	296.83 ± 39.81^a^	1.31 ± 1.03^a^
Significance level	**	**	**	**	**	**
**Storage time**						
0	52.08 ± 14.17^d^	51.39 ± 13.70^b^	216.21 ± 44.49^a^	230.83 ± 46.53^a^	267.50 ± 61.08^a^	2.93 ± 0.18^a^
1st month	48.75 ± 17.06^e^	56.22 ± 17.14^a^	163.77 ± 31.34^c^	191.68 ± 49.03^b^	201.79 ± 57.17^e^	1.84 ± 0.49^b^
3rd month	52.95 ± 15.91^cd^	43.67 ± 14.87^c^	170.70 ± 26.85^b^	148.45 ± 28.15^f^	231.57 ± 48.08^c^	1.34 ± 0.59^c^
6th month	53.52 ± 12.58^c^	41.48 ± 11.93^d^	151.08 ± 25.93^e^	171.37 ± 31.82^c^	210.70 ± 43.63^d^	0.50 ± 0.33^d^
9th month	54.96 ± 14.73^b^	41.22 ± 15.38^d^	100.64 ± 29.64^f^	156.25 ± 34.19^d^	205.75 ± 47.79^e^	0.36 ± 0.26^e^
12th month	63.00 ± 15.60^a^	40.04 ± 16.46^e^	157.88 ± 26.15^d^	153.67 ± 33.20^e^	254.04 ± 58.31^b^	0.22 ± 0.15^f^
Significance level	**	**	**	**	**	**
**Storage temperature**						
25°C ± 2°C	54.13 ± 15.71^a^	44.45 ± 16.16^b^	155.53 ± 47.06^b^	177.47 ± 43.72^a^	223.17 ± 61.05^b^	0.95 ± 1.02^b^
4°C ± 2°C	54.28 ± 15.47^a^	46.90 ± 15.91^a^	164.57 ± 44.77^a^	173.28 ± 50.72^b^	233.95 ± 54.97^a^	1.45 ± 0.98^a^
Significance level	NS	**	**	**	**	**
**Packaging type**						
Plastic	54.29 ± 15.70^a^	45.74 ± 15.93^a^	158.35 ± 46.82^b^	175.84 ± 46.78	225.57 ± 58.68^b^	1.15 ± 1.07^b^
Glass	54.23 ± 15.50^a^	45.75 ± 16.19^a^	162.32 ± 45.00^a^	175.29 ± 47.95	232.01 ± 57.73^a^	1.26 ± 0.99^a^
Significance level	NS	NS	**	NS	**	**
S × STi	**	**	**	**	**	**
S × STe	NS	NS	**	**	*	*
S × *P*	NS	NS	*	**	**	*
STi × STe	NS	**	**	**	**	**
STi × *P*	**	NS	**	**	**	**
STe × *P*	**	NS	**	**	NS	NS

*Note:* Different superscript letters (a–e) within the same column indicate significant differences (*p* < 0.01). Units are expressed as mg GAE/100 mL (TPC), mg QE/100 mL (TFC), μM TE/100 mL (DPPH, ABTS, and FRAP), and mg C3G/100 mL (TMA).

Abbreviations: C, Control group; NS, Not Significant; P, Packaging Type; PGly‐BB, Propolis glycerol–water extract enriched blueberry beverage; PPG‐BB, Propolis propylene glycol–water extract enriched blueberry beverage; PW‐BB, Propolis water extract enriched blueberry beverage; S, Sample Type; STe, Storage Temperature; STi, Storage Time.

Sample type, storage time, and their interaction significantly affected TPC, TFC, TMA, and all antioxidant activity (DPPH, ABTS, and FRAP) results (*p* < 0.01). The significant interaction between sample type and storage time demonstrates that the effects of storage were not uniform across the beverage groups. As storage time increased, a nonlinear decrease was observed in TFC, TMA, and antioxidant activity. In particular, a decrease of approximately 93% in anthocyanins from the beginning to the 12th month was noteworthy. However, partial increases in TPC values (ranging from 52.95 to 63 mg GAE/100 mL) were observed after the third month. Partial increases in DPPH and FRAP values were also observed during the later stages of storage. These changes may be associated with storage‐related modifications occurring within the beverage matrix, which could affect the formation or availability of compounds exhibiting antioxidant activity and reactivity toward the Folin–Ciocalteu reagent. However, the specific chemical transformations responsible for these observations were not investigated in the present study. The TPC analysis performed using the Folin–Ciocalteu method is a total measurement, not focused on individual compounds. It should also be noted that antioxidant activity in complex beverage systems cannot be attributed exclusively to phenolic compounds, since storage‐related degradation products and interactions among beverage constituents may also contribute to the measured responses. Therefore, the higher phenolic levels observed in beverages containing propylene glycol–water extract should not be interpreted as direct evidence of enhanced preservation. Furthermore, many other non‐phenolic compounds that may form during this process, such as reducible sugars and certain amino acids, may also react with the Folin–Ciocalteu reagent, thereby increasing the TPC value (Torres et al. 2024). Consistent with our findings, Vasilaki et al. (2019) reported that antioxidant activity in carbonated orange juices prepared with propolis extract exhibited variable trends over a 4‐month storage period. Researchers noted that samples initially exhibited high radical scavenging activity, but a significant decrease occurred during the early stages of storage, and antioxidant activity became relatively stable in later stages.

Samples stored under refrigeration showed significantly higher TFC, DPPH, and TMA values (*p* < 0.01) than those stored at room temperature (Table 2). This is thought to be related to the fact that low‐temperature storage slows the degradation of compounds that exhibit antioxidant activity, such as flavonoids and anthocyanins, which are sensitive to oxidation. Our findings are consistent with previous studies showing that various antioxidants and bioactive compounds are preserved in beverages at low storage temperatures (Chi et al. 2025; La Torre et al. 2021; Orellana‐Palma et al. 2020). However, Vasilaki et al. (2019) reported that storage temperature (4°C, 25°C, and 45°C) had no significant effect on the antioxidant activity of the beverages and that stability varied considerably with storage period. The difference between the mentioned study and our findings indicates that the effect of storage temperature on beverages is not always consistent. This situation is caused by the unique chemical composition of beverages, which is influenced by the origin of the propolis, the extraction solvent, and the carrier matrix (fruit juice). Our findings also showed that the interaction between storage time and temperature was statistically significant (*p* < 0.01) in all analyses except TPC. This indicates that the effects of storage temperature on antioxidant‐active compounds depended on storage duration.

The type of packaging had a limited effect on the TPC and TFC contents of propolis‐containing beverages (*p* > 0.05). In other words, the total amounts of phenolic and flavonoid compounds were preserved similarly regardless of packaging types during storage. However, we should note that PPG‐BB group samples stored in glass containers showed the highest TPC values among all groups (0‐ and 12th‐month data were 74.62 and 90.02 mg GAE/100 mL, respectively, data not shown). Consistent with our findings, Saarniit et al. (2023) reported that, in their study on the stability of phenolic compounds in fruit, berry, and plant purees, the TPC level was independent of the packaging material, whereas the observed changes were primarily dependent on the chemical composition of the product and the storage conditions. Bratu et al. (2021) determined TPC values in commercial beers packaged in glass, plastic, and aluminum containers as 170.43 ± 10.09, 160 ± 8.56, and 164.74 ± 12.04 mg GAE/L, respectively. The study concluded that packaging material was not a significant factor affecting TPC. Glass packaging was more advantageous in terms of DPPH radical scavenging capacity and reducing power (FRAP) (Table 2). The effect of packaging type on ABTS values was limited (*p* > 0.05). Although both methods (DPPH and ABTS) indicate free radical scavenging activity, the differences in their findings result from different radical systems. Additionally, the DPPH analysis is sensitive to lipophilic and moderately polar compounds, while the ABTS analysis is primarily sensitive to hydrophilic and highly polar compounds, which also contributes to the observed differences. As in our study, the effectiveness of glass packaging in preserving antioxidant compounds has also been reported for various polyphenol‐rich fruit‐based beverages (Muhamad et al. 2022; Zouaoui et al. 2024).

Anthocyanins are highly sensitive to light, oxygen, and pH changes. These compounds can lose their effects during storage by degradation or polymerization. The current study found that propolis‐containing blueberry beverages stored in glass packaging had statistically higher levels of monomeric anthocyanins than those stored in plastic packaging (*p* < 0.01). The TMA content of beverages stored in glass was found to be 1.26 mg C3G/100 mL on average, whereas it was 1.15 mg C3G/100 mL in plastic. The improved oxygen barrier properties of glass packaging may have slowed anthocyanin degradation in propolis extract‐containing blueberry beverages stored in glass. Similar to our findings, a study assessing the stability of anthocyanins in red cabbage‐roselle mixed beverages during storage in glass, polypropylene, and tin packages reported that glass packaging was superior (Muhamad et al. 2022). Researchers determined that the total anthocyanin level, initially approximately 263 mg/L, decreased to 34.36, 10.44, and 30.50 mg/L in glass, polypropylene, and tin packaging, respectively, at the end of the 6th month. Accordingly, glass bottles preserved approximately 13% of the anthocyanins, whereas only 4% remained in plastic bottles.

### 3.4 Changes in the Phenolic Profile

The stability of phenolic compounds varies significantly depending on storage conditions and packaging type (Singh et al. 2020). This study aimed to determine changes in phenolic compound content in beverages containing propolis extracts prepared with different solvents, stored in plastic packaging at different temperatures (room and refrigerator temperatures) for 12 months. Changes in the phenolic profile of the beverages are presented in the Table S2. Phenolic profile analyses revealed the presence of gallic acid, epigallocatechin, chlorogenic acid, catechin, epicatechin, caffeic acid, rutin, *p*‐coumaric acid, trans‐sinapic acid, ferulic acid, myricetin, resveratrol, luteolin, quercetin, apigenin, diosmetin, sinensetin, and chrysin. However, only caffeic acid, rutin, *p*‐coumaric acid, ferulic acid, resveratrol, apigenin, and chrysin were detected in the beverages. Considering the sample, storage temperature, packaging type, and storage time, the amounts of caffeic acid were determined to range from 0.32 to 11.32 mg/L, rutin from 1.95 to 5.32 mg/L, *p*‐coumaric acid from 4.09 to 13.20 mg/L, ferulic acid from 3.56 to 18.53 mg/L, resveratrol from 5.93 to 14.16 mg/L, apigenin from 1.89 to 1.96 mg/L, and chrysin from 1.58 to 13.51 mg/L. The phenolic compounds detected in all beverages were caffeic acid, rutin, and resveratrol. Resveratrol was the compound detected at the highest levels (5.93–14.16 mg/L) in all beverages.

In the C group, caffeic acid, rutin, and resveratrol could be detected, while *p*‐coumaric acid, ferulic acid, apigenin, and chrysin could not be detected. In PW‐BB, caffeic acid, rutin, *p*‐coumaric acid, ferulic acid, and resveratrol could be detected, but apigenin and chrysin could not be detected. In PGly‐BB, caffeic acid, rutin, *p*‐coumaric acid, ferulic acid, resveratrol, and chrysin could be detected, but apigenin was the only compound that could not be detected. In PPG‐BB, all 7 detectable compounds (caffeic acid, rutin, *p*‐coumaric acid, ferulic acid, resveratrol, apigenin, and chrysin) were detected in varying amounts. At this point, it is observed that the phenolic compounds from the solvents used for propolis extracts in beverages differ. Our previous study conducted at the extract level (Sengul et al. 2026) demonstrated that solvent polarity significantly affects the phenolic composition of propolis. Current findings indicate that these solvent‐dependent compositional differences persist after incorporation into the beverage matrix and during storage. The beverages containing PPG extract exhibited a broader detectable phenolic profile and higher concentrations of several phenolic compounds than the other beverage groups. A significant reduction was observed in most components in the C group and PW‐BB. However, the mechanisms responsible for these changes were not investigated in the present study; therefore, the observed differences should be interpreted primarily as compositional variations among beverage groups. Similar storage‐related changes in phenolic compounds and antioxidant properties have previously been reported in propolis‐containing beverages (Vasilaki et al. 2019). Better stability was observed in PGly‐BB. Compared with PW‐BB, PGly‐BB samples generally retained more detectable phenolic compounds throughout storage. PPG‐BB was the group in which the smallest changes in phenolic composition were observed, and relative increases in the concentrations of some compounds were detected during storage. These findings suggest that solvent type was an important factor influencing the phenolic profile of blueberry beverages during long‐term storage.

The phenolic compound profiles of all beverages varied with storage temperature, packaging type, and storage time. The stability of the compounds also changed under these conditions. At room temperature (25°C ± 2°C), most components decreased over time. This indicates that high temperatures cause the degradation or oxidation of the components. Samples stored in the refrigerator were more stable in component values. However, even in the refrigerator, some components decreased over time. Nevertheless, such low temperatures can help reduce the rate of component degradation. While storage at room temperature decreases compound stability over time across different beverages, storage at low temperatures, such as in a refrigerator, better preserves compound stability (Chi et al. 2025; Wojdylo et al. 2019).

The analysis results show that packaging type has a significant impact on component stability. Plastic packaging shows a greater reduction, particularly in rutin. Plastic materials can generally lead to component degradation due to their oxygen permeability. It has been observed that the compound is more stable in glass packaging. The lower oxygen permeability of glass packaging may contribute to improved stability of these compounds. In terms of maintaining stability, it can be said that it provides protective effects for compounds other than rutin. The literature indicates that plastic packaging can cause component degradation due to its oxygen permeability, whereas glass packaging offers better control over this. In their study, Pipliya et al. (2024) reported that glass bottles were superior to PET bottles in preserving pineapple juice quality during storage.

When comparing the differences between the beginning and 12 months, a general decrease was observed in the amounts of rutin, apigenin, and chrysin, while the amounts of caffeic acid, *p*‐coumaric acid, ferulic acid, and resveratrol remained stable. This suggests that oxidation, hydrolysis, or other chemical reactions contribute to the reduction of these phenolic compounds (Chang et al. 2006). Furthermore, interactions between phenolic compounds or between phenolics and other components such as dietary fiber are another important factor influencing the rate of phenolic degradation (Zhou et al. 2025).

### 3.5 Correlation Analysis and Discrimination of the Blueberry Beverages With Principal Component Analysis (PCA)

PCA was applied to the blueberry beverages dataset, which included multiple dependent and independent variables (TPC, TFC, ABTS, DPPH, FRAP, TMA, individual phenolics, packaging type, storage temperature, storage time, and sample type). This method made it easier to visualize similarities and differences between samples and relationships among variables, and to explain the fundamental structure of the dataset by reducing it to a small number of components.

**FIGURE 3:**
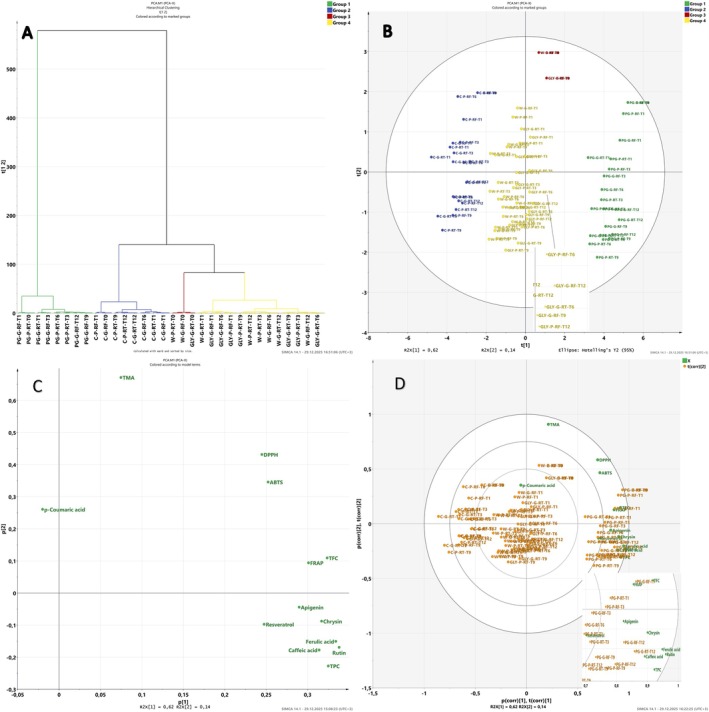
Hierarchical cluster analysis (A), score scatter plot (B), loading scatter plot (C), and biplot (D) of the principal component analysis of blueberry beverages. C, Control group (blueberry beverage without propolis); G, Glass bottle; GLY, Blueberry beverages with aqueous glycerol extract; P, Plastic bottle; PG, Blueberry beverages with aqueous propylene glycol extract; RF, Refrigerated; RT, Room temperature; TO–T12, Storage time between 0 and 12 months; W, Blueberry beverages with aqueous extract.

Hierarchical cluster analysis (HCA) of PCA results, score scatter plot, loading scatter plot, and biplot are shown in Figure 3A–D. The first two principal components (PC1 = 62% and PC2 = 14%) account for 76% of the variation among propolis‐containing blueberry beverages. The HCA diagram showed that beverage samples were clearly separated into distinct clusters based on TPC, TFC, TMA, antioxidant parameters (DPPH, ABTS, and FRAP), and individual phenolic profiles (Figure 3). The PPG‐BB and control groups were clearly separated from the other groups, whereas the PW‐BB and PGly‐BB groups showed similar characteristics across the analyzed parameters.

The score scatter plot (Figure 3B) supported HCA, showing that the beverages were divided into four main groups. The sample type had a significant effect on clustering in the relevant plot. PPG‐BB samples clustered in the positive direction of PC1 and were distinctly separated from the control group. This separation indicates that the addition of the propylene glycol extract affects the overall biological activity profile of the samples. Although samples containing water and glycerol extracts were located close to each other, in general, PW‐BB samples were more to the left of PC1, while PGly‐BB samples were in the middle and right of PC1. In particular, PGly‐BB samples refrigerated in glass bottles clustered to the right of PC1. Additionally, the samples showed separation by storage period. In general, samples stored for the first 3 months clustered on the positive side of PC2, while samples stored for longer periods were on the opposite side. This distribution suggests that storage duration contributed to sample discrimination independently of extract type, indicating progressive changes in the measured compositional and functional attributes throughout storage.

**FIGURE 4:**
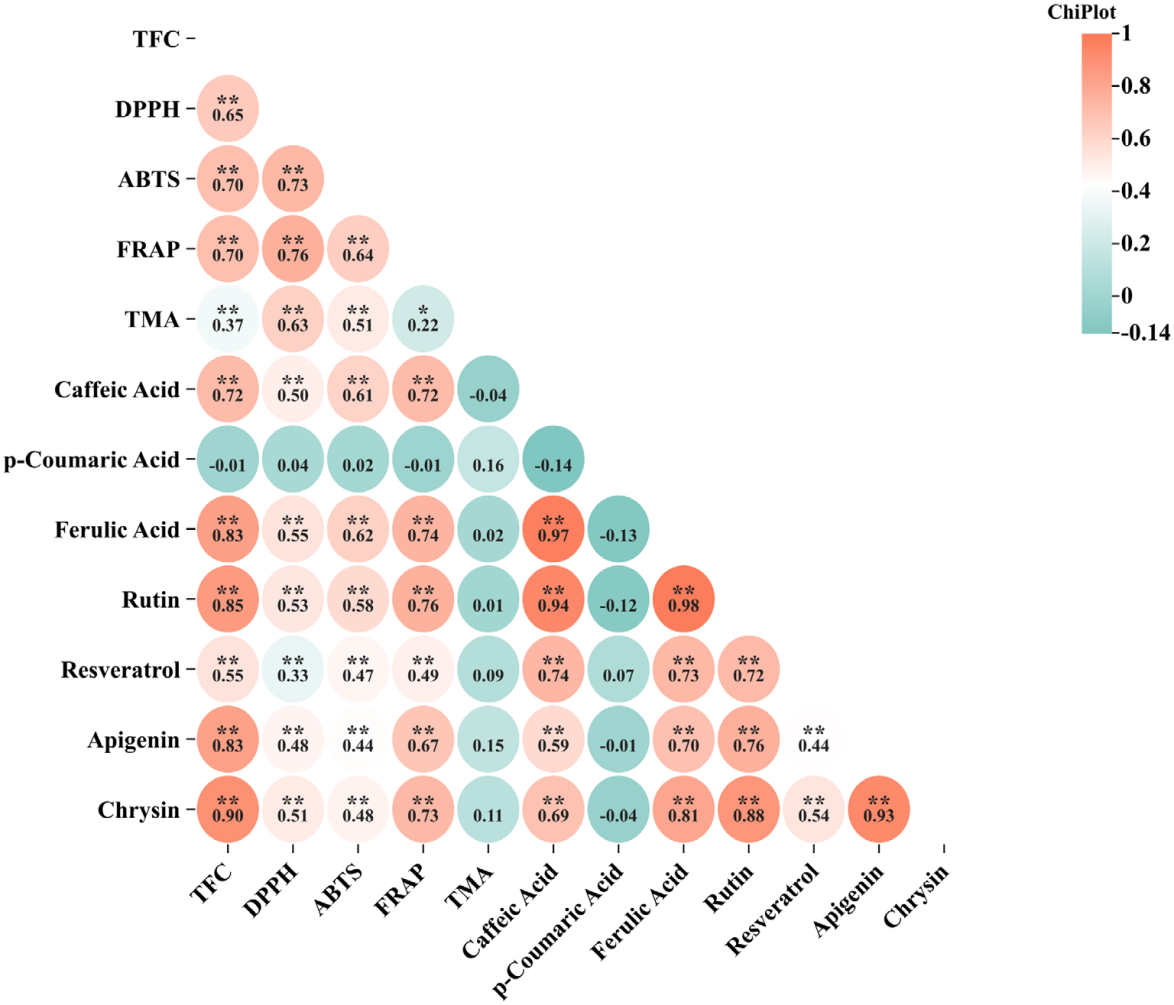
Correlation matrix of blueberry beverages data. In the graph, bubble size indicates the degree of correlation, while color transitions indicate the direction of the relationship, ranging from orange (positive) to blue (negative).

In the loading scatter plot, the two groups DPPH, FRAP, ABTS, and TFC, along with apigenin, chrysin, resveratrol, ferulic acid, caffeic acid, rutin, and TPC, which are located in the positive direction of PC1, showed a high level of positive correlation among themselves (*p* < 0.01) (Figures 3C and 4). Collectively, the distribution of these variables along PC1 indicates that this component was primarily associated with phenolic composition and antioxidant‐related characteristics. Therefore, the separation of samples along the horizontal axis appears to reflect differences in their overall phenolic and antioxidant profiles. TMA, positioned on the positive side of PC2, had a statistically significant effect (*p* < 0.01) on TFC and antioxidant activity (DPPH and ABTS); however, it was located far from individual non‐anthocyanin phenolics and did not correlate with them. The separate positioning of *p*‐coumaric acid in the negative direction of PC1 suggests that, in contrast to apigenin, chrysin, resveratrol, ferulic acid, caffeic acid, and rutin, its contribution to overall antioxidant capacity is minimal (Figure 3C). This relationship is further supported by the heat map (Figure 4).

In the biplot (Figure 3D), the close proximity of PPG‐BB samples to apigenin, chrysin, resveratrol, ferulic acid, caffeic acid, and rutin indicates a stronger association between these compounds and the PPG‐BB samples. The similar positioning of beverages within the same group in antioxidant activity parameters (ABTS, DPPH, and FRAP) suggests that these samples have a rich profile of individual phenolic compounds (Table S2) and that these compounds may contribute substantially to antioxidant capacity. The close association of these variables within the same region of the biplot further indicates that sample discrimination was driven by the combined variation of multiple phenolic and antioxidant‐related parameters rather than by a single dominant variable. The clustering behavior observed in the current study parallels the solvent‐dependent discrimination reported in the extract‐focused investigation (Sengul et al. 2026), confirming that solvent‐induced compositional differences influence the complex fruit‐based beverage system.

## 4 Conclusion

This study comprehensively evaluated the changes in quality characteristics of blueberry‐based beverages produced by adding propolis extracts prepared with different solvents (water, glycerol, and propylene glycol) under different packaging types (glass and PET) and storage conditions (4°C ± 2°C and 25°C ± 2°C) over 12 months. The results revealed that the type of solvent used in the propolis extract, as well as the storage time, temperature, and packaging type, had statistically significant effects on the physicochemical properties, bioactive component content, antioxidant capacity, and phenolic profiles of the beverages.

In particular, the PPG‐BB group exhibited superior performance compared to other groups in TPC, TFC, TMA, and antioxidant activity as measured by DPPH, ABTS, and FRAP methods. The PPG‐BB group consistently exhibited higher levels of phenolic compounds and antioxidant activity than the other beverage groups throughout storage. Phenolic profile analyses also support these findings, and the detection of all phenolic compounds examined (caffeic acid, rutin, *p*‐coumaric acid, ferulic acid, resveratrol, apigenin, and chrysin) could be detected only in the PPG‐BB group, demonstrating that solvent selection is a critical factor in functional beverage formulations.

Consequently, the combination of propylene glycol‐based propolis extract and glass packaging was the most effective in maintaining the phenolic profile, antioxidant activity, and physicochemical properties evaluated in this study during storage. These findings provide noteworthy scientific contributions to the development of functional beverages. Since microbial stability and food safety parameters were not evaluated, the conclusions of this study are limited to the physicochemical properties, phenolic profile, and antioxidant characteristics investigated. Another limitation is that sensory evaluation was conducted only on freshly prepared beverages and was not repeated during storage; therefore, changes in sensory attributes over the storage period could not be assessed. Future studies should include microbial stability, rheological behavior, sedimentation characteristics, dissolved oxygen measurements, and degradation kinetics to provide a more comprehensive evaluation of product quality changes during storage and the practical applicability of propolis‐enriched blueberry beverages.

## Author Contributions

**Elif Feyza Topdas:** investigation, writing – original draft, writing – review and editing, methodology. **İsa Arslan Karakutuk:** investigation, visualization, methodology, formal analysis, software, data curation, validation. **Seda Ufuk:** investigation, writing – original draft, writing – review and editing, methodology. **Sefa Aksoy:** formal analysis, methodology. **Melek Zor:** investigation, writing – original draft, writing – review and editing, methodology. **Memnune Sengul:** conceptualization, funding acquisition, project administration, supervision, resources, methodology.

## Funding

Funding for this research was provided by Atatürk University BAP Unit within the Research Universities Support Program (Grant No. FBA‐2023‐8491).

## Conflicts of Interest

The authors declare no conflicts of interest.

## Supporting information


**Table S1:** Phenolic profile and RP‐HPLC‐UV analysis parameters of blueberry beverages.
**Table S2:** Effect of sample type, storage temperature, packaging type, and storage time on caffeic acid, rutin, *p*‐coumaric acid, ferulic acid, resveratrol, apigenin, and chrysin content (mg/L) of beverages.

## Data Availability

The data that support the findings of this study are available from the corresponding author upon reasonable request.
