# Supplementary material for: Storage‐Induced Changes in Phenolic Profile and Antioxidant Capacity of Functional Blueberry Beverages Enriched With Propolis
Source: Food Sci Nutr. 2026 Aug 16;14(8):e72266. doi: 10.1002/fsn3.72266 (PMC13478555; doi:10.1002/fsn3.72266)
Supplement: Supplementary file 1 [file FSN3-14-e72266-s001.docx]

**Table S1.** Phenolic profile and RP-HPLC-UV analysis parameters of blueberry beverages

| Compound | RT | Equation | R^2^ | LR | LOD | LOQ |
| --- | --- | --- | --- | --- | --- | --- |
|  |  |  |  |  |  |  |
| Gallic acid | 10.103 | y = 23.355x + -1.102 | 1.00000 | 1-50 | 0.02 | 0.07 |
| Epigallocatechin | 15.364 | y = 1.895x + 0.331 | 0.99993 | 1-50 | 0.21 | 0.63 |
| Chlorogenic acid | 16.128 | y = 12.257x + 0.426 | 0.99999 | 1-50 | 0.07 | 0.21 |
| Catechin | 16.409 | y = 6.376x + 3.208 | 0.99981 | 1-50 | 0.10 | 0.30 |
| Epicatechin | 17.406 | y = 5.746x + 0.217 | 0.99999 | 1-50 | 0.04 | 0.13 |
| Caffeic acid | 17.754 | y = 26.078x + 4.284 | 0.99998 | 1-50 | 0.02 | 0.06 |
| Rutin | 18.317 | y = 6.766x + 0.839 | 0.99999 | 1-50 | 0.07 | 0.20 |
| *p*-Coumaric acid | 19.888 | y = 39.386x + 3.417 | 0.99999 | 1-50 | 0.02 | 0.07 |
| Trans-sinapic acid | 20.173 | y = 10.658x + 1.108 | 0.99998 | 1-50 | 0.04 | 0.12 |
| Ferulic acid | 20.457 | y = 23.966x + 0.344 | 0.99998 | 1-50 | 0.02 | 0.06 |
| Myricetin | 21.646 | y = 10.658x + -1.147 | 0.99998 | 1-50 | 0.36 | 1.09 |
| Resveratrol | 23.003 | y = 30.513x + 1.585 | 0.99999 | 1-50 | 0.01 | 0.04 |
| Luteolin | 23.657 | y = 14.926x + 0.163 | 0.99999 | 1-50 | 0.04 | 0.13 |
| Quercetin | 23.885 | y = 12.546x + -0.213 | 1.00000 | 1-50 | 0.31 | 0.93 |
| Apigenin | 25.309 | y = 19.761x + 2.660 | 0.99999 | 1-50 | 0.17 | 0.52 |
| Diosmetin | 25.658 | y = 19.245x + 2.326 | 0.99999 | 1-50 | 0.15 | 0.46 |
| Sinensetin | 27.963 | y = 12.067x + -0.026 | 0.99999 | 1-50 | 0.07 | 0.20 |
| Chrysin | 30.653 | y = 27.932x + 0.941 | 0.99999 | 1-50 | 0.01 | 0.04 |

*RT: Retention time (min); R^2^: Correlation coefficient; LR: Linear range µg/mL; LOD: Limit of detection (µg/mL); LOQ: Limit of quantification (µg/mL)

**Table S2.** Effect of sample type, storage temperature, packaging type, and storage time on caffeic acid, rutin, *p*-coumaric acid, ferulic acid, resveratrol, apigenin, and chrysin content (mg/L) of beverages

| Sample | Storage temperature | Packaging type | Storage time | Phenolic compounds | | | | | | |
| --- | --- | --- | --- | --- | --- | --- | --- | --- | --- | --- |
|  |  |  |  | Caffeic acid | Rutin | *p*-Coumaric acid | Ferulic acid | Resveratrol | Apigenin | Chrysin |
| C | 25±2 °C | Plastic | 0 | 1.31 | 4.76 | ND | ND | 11.77 | ND | ND |
|  |  |  | 1st Month | 0.60 | 2.91 | ND | ND | 9.04 | ND | ND |
|  |  |  | 3rd Month | 0.67 | 2.57 | ND | ND | 9.06 | ND | ND |
|  |  |  | 6th Month | 0.92 | 2.57 | ND | ND | 10.71 | ND | ND |
|  |  |  | 9th Month | 0.89 | 2.55 | ND | ND | 10.50 | ND | ND |
|  |  |  | 12th Month | 1.08 | 3.48 | ND | ND | 10.97 | ND | ND |
|  |  | Glass | 0 | 1.31 | 4.76 | ND | ND | 11.77 | ND | ND |
|  |  |  | 1st Month | 0.32 | 1.95 | ND | ND | 5.93 | ND | ND |
|  |  |  | 3rd Month | 0.90 | 2.52 | ND | ND | 8.82 | ND | ND |
|  |  |  | 6th Month | 1.16 | 2.91 | ND | ND | 10.64 | ND | ND |
|  |  |  | 9th Month | 0.76 | 2.43 | ND | ND | 8.76 | ND | ND |
|  |  |  | 12th Month | 1.28 | 2.67 | ND | ND | 9.80 | ND | ND |
|  | 4±2 °C | Plastic | 0 | 1.31 | 4.76 | ND | ND | 11.77 | ND | ND |
|  |  |  | 1st Month | 0.82 | 4.12 | ND | ND | 10.17 | ND | ND |
|  |  |  | 3rd Month | 0.92 | 4.40 | ND | ND | 10.51 | ND | ND |
|  |  |  | 6th Month | 0.93 | 4.10 | ND | ND | 11.21 | ND | ND |
|  |  |  | 9th Month | 0.84 | 3.35 | ND | ND | 10.92 | ND | ND |
|  |  |  | 12th Month | 1.15 | 3.07 | ND | ND | 10.68 | ND | ND |
|  |  | Glass | 0 | 1.31 | 4.76 | ND | ND | 11.77 | ND | ND |
|  |  |  | 1st Month | 0.82 | 3.82 | ND | ND | 10.02 | ND | ND |
|  |  |  | 3rd Month | 0.86 | 3.74 | ND | ND | 10.15 | ND | ND |
|  |  |  | 6th Month | 0.87 | 4.57 | ND | ND | 10.93 | ND | ND |
|  |  |  | 9th Month | 0.95 | 3.40 | ND | ND | 10.23 | ND | ND |
|  |  |  | 12th Month | 1.13 | 3.32 | ND | ND | 10.18 | ND | ND |
| PW-BB | 25±2 °C | Plastic | 0 | 6.25 | 4.86 | 5.54 | 5.05 | 11.47 | ND | ND |
|  |  |  | 1st Month | 4.00 | 2.47 | 4.09 | 3.72 | 9.89 | ND | ND |
|  |  |  | 3rd Month | 5.04 | 2.42 | 4.63 | 4.00 | 10.02 | ND | ND |
|  |  |  | 6th Month | 5.95 | 3.05 | 5.69 | 5.49 | 12.52 | ND | ND |
|  |  |  | 9th Month | 5.81 | 2.98 | 5.26 | 4.90 | 11.66 | ND | ND |
|  |  |  | 12th Month | 5.98 | 2.84 | 5.37 | 5.00 | 10.83 | ND | ND |
|  |  | Glass | 0 | 6.25 | 4.86 | 5.54 | 5.05 | 11.47 | ND | ND |
|  |  |  | 1st Month | 4.31 | 2.70 | 4.37 | 4.07 | 10.60 | ND | ND |
|  |  |  | 3rd Month | 5.16 | 2.72 | 4.94 | 5.05 | 10.94 | ND | ND |
|  |  |  | 6th Month | 6.68 | 3.09 | 5.91 | 5.59 | 12.38 | ND | ND |
|  |  |  | 9th Month | 5.92 | 2.90 | 5.30 | 5.07 | 11.06 | ND | ND |
|  |  |  | 12th Month | 6.06 | 2.91 | 5.40 | 5.21 | 10.07 | ND | ND |
|  | 4±2 °C | Plastic | 0 | 6.25 | 4.86 | 5.54 | 5.05 | 11.47 | ND | ND |
|  |  |  | 1st Month | 4.90 | 4.71 | 5.24 | 3.56 | 12.19 | ND | ND |
|  |  |  | 3rd Month | 5.39 | 4.25 | 5.40 | 5.10 | 11.91 | ND | ND |
|  |  |  | 6th Month | 6.25 | 3.25 | 5.85 | 5.62 | 11.48 | ND | ND |
|  |  |  | 9th Month | 7.41 | 3.35 | 5.94 | 5.57 | 12.26 | ND | ND |
|  |  |  | 12th Month | 6.69 | 3.39 | 6.32 | 7.09 | 12.04 | ND | ND |
|  |  | Glass | 0 | 6.25 | 4.86 | 5.54 | 5.05 | 11.47 | ND | ND |
|  |  |  | 1st Month | 5.79 | 4.91 | 5.74 | 5.26 | 11.49 | ND | ND |
|  |  |  | 3rd Month | 5.42 | 4.27 | 5.38 | 5.12 | 11.86 | ND | ND |
|  |  |  | 6th Month | 5.72 | 3.00 | 5.44 | 5.07 | 11.30 | ND | ND |
|  |  |  | 9th Month | 6.39 | 3.27 | 6.13 | 5.71 | 12.38 | ND | ND |
|  |  |  | 12th Month | 7.91 | 3.20 | 5.80 | 5.86 | 11.30 | ND | ND |
| PGly-BB | 25±2 °C | Plastic | 0 | 7.04 | 4.11 | 7.19 | 7.53 | 11.66 | ND | 1.58 |
|  |  |  | 1st Month | 4.68 | 2.81 | 6.23 | 6.46 | 11.37 | ND | 1.68 |
|  |  |  | 3rd Month | 4.94 | 2.36 | 6.05 | 6.50 | 11.06 | ND | 1.34 |
|  |  |  | 6th Month | 7.42 | 2.79 | 7.57 | 9.42 | 12.71 | ND | 1.48 |
|  |  |  | 9th Month | 6.51 | 2.55 | 6.80 | 7.10 | 11.94 | ND | 1.33 |
|  |  |  | 12th Month | 6.38 | 2.56 | 6.45 | 6.67 | 10.93 | ND | 1.28 |
|  |  | Glass | 0 | 7.04 | 4.11 | 7.19 | 7.53 | 11.66 | ND | 1.58 |
|  |  |  | 1st Month | 4.54 | 3.01 | 5.97 | 6.19 | 10.93 | ND | 1.41 |
|  |  |  | 3rd Month | 6.21 | 2.90 | 6.33 | 6.85 | 11.25 | ND | 1.36 |
|  |  |  | 6th Month | 7.47 | 2.76 | 7.62 | 9.31 | 12.92 | ND | 1.45 |
|  |  |  | 9th Month | 7.16 | 2.73 | 7.49 | 9.08 | 12.45 | ND | 1.51 |
|  |  |  | 12th Month | 6.51 | 2.52 | 6.59 | 6.96 | 10.44 | ND | 1.21 |
|  | 4±2 °C | Plastic | 0 | 7.04 | 4.11 | 7.19 | 7.53 | 11.66 | ND | 1.58 |
|  |  |  | 1st Month | 5.64 | 3.59 | 7.33 | 7.62 | 12.28 | ND | 1.57 |
|  |  |  | 3rd Month | 5.53 | 3.19 | 7.03 | 7.45 | 11.85 | ND | 1.54 |
|  |  |  | 6th Month | 7.14 | 3.40 | 7.58 | 9.11 | 12.02 | ND | 1.49 |
|  |  |  | 9th Month | 6.09 | 3.45 | 7.63 | 8.23 | 12.54 | ND | 1.53 |
|  |  |  | 12th Month | 7.77 | 3.35 | 7.76 | 8.53 | 11.56 | ND | 1.56 |
|  |  | Glass | 0 | 7.04 | 4.11 | 7.19 | 7.53 | 11.66 | ND | 1.58 |
|  |  |  | 1st Month | 5.40 | 3.71 | 7.06 | 7.33 | 11.30 | ND | 1.48 |
|  |  |  | 3rd Month | 6.81 | 3.28 | 7.25 | 7.79 | 11.67 | ND | 1.59 |
|  |  |  | 6th Month | 6.94 | 3.06 | 7.22 | 8.68 | 11.69 | ND | 1.47 |
|  |  |  | 9th Month | 7.46 | 3.61 | 7.70 | 9.17 | 12.24 | ND | 1.59 |
|  |  |  | 12th Month | 7.44 | 3.04 | 6.92 | 9.33 | 11.65 | ND | 1.44 |
| PPG-BB | 25±2 °C | Plastic | 0 | 8.62 | 5.32 | 11.37 | 14.83 | 12.58 | 1.89 | 12.41 |
|  |  |  | 1st Month | 8.09 | 2.93 | 10.61 | 13.33 | 12.03 | 1.77 | 11.42 |
|  |  |  | 3rd Month | 8.79 | 2.70 | 10.85 | 14.07 | 11.99 | 1.68 | 10.90 |
|  |  |  | 6th Month | 10.99 | 3.01 | 12.77 | 16.58 | 14.16 | 0.56 | 7.72 |
|  |  |  | 9th Month | 9.91 | 3.03 | 11.79 | 15.04 | 12.99 | 1.69 | 10.78 |
|  |  |  | 12th Month | 9.80 | 3.01 | 11.39 | 14.34 | 11.66 | 1.65 | 10.30 |
|  |  | Glass | 0 | 8.62 | 5.32 | 11.37 | 14.83 | 12.58 | 1.89 | 12.41 |
|  |  |  | 1st Month | 7.57 | 2.82 | 9.93 | 12.39 | 10.98 | 0.65 | 10.74 |
|  |  |  | 3rd Month | 9.23 | 2.72 | 11.24 | 14.65 | 12.30 | 1.81 | 11.45 |
|  |  |  | 6th Month | 11.32 | 3.28 | 13.20 | 17.39 | 13.55 | 0.52 | 7.12 |
|  |  |  | 9th Month | 10.46 | 3.24 | 12.21 | 15.87 | 12.48 | 1.79 | 11.33 |
|  |  |  | 12th Month | 10.57 | 3.10 | 12.05 | 15.63 | 11.18 | 1.82 | 11.34 |
|  | 4±2 °C | Plastic | 0 | 8.62 | 5.32 | 11.37 | 14.83 | 12.58 | 1.89 | 12.41 |
|  |  |  | 1st Month | 8.25 | 3.58 | 11.03 | 14.33 | 12.05 | 1.83 | 12.30 |
|  |  |  | 3rd Month | 9.12 | 3.86 | 12.36 | 16.09 | 13.07 | 0.80 | 13.51 |
|  |  |  | 6th Month | 9.12 | 3.26 | 11.96 | 15.82 | 12.75 | 0.57 | 8.95 |
|  |  |  | 9th Month | 9.37 | 3.30 | 12.35 | 16.14 | 13.44 | 1.75 | 12.13 |
|  |  |  | 12th Month | 10.00 | 2.91 | 12.63 | 16.81 | 12.94 | 0.83 | 12.68 |
|  |  | Glass | 0 | 8.62 | 5.32 | 11.37 | 14.83 | 12.58 | 1.89 | 12.41 |
|  |  |  | 1st Month | 7.45 | 4.09 | 11.85 | 15.38 | 12.78 | 1.96 | 12.84 |
|  |  |  | 3rd Month | 8.39 | 3.47 | 11.48 | 14.62 | 12.68 | 1.91 | 13.02 |
|  |  |  | 6th Month | 9.19 | 3.15 | 11.67 | 15.34 | 12.44 | 1.35 | 8.54 |
|  |  |  | 9th Month | 9.62 | 3.38 | 12.58 | 18.53 | 13.97 | 0.79 | 12.43 |
|  |  |  | 12th Month | 9.56 | 2.83 | 11.75 | 16.16 | 12.31 | 0.80 | 12.07 |

C: Control group, PW-BB: Propolis water extract–enriched blueberry beverage, PGly-BB: Propolis glycerol-water extract–enriched blueberry beverage, PPG-BB: Propolis propylene glycol–water extract–enriched blueberry beverage, S: Sample Type, STi: Storage Time, STe: Storage Temperature, P: Packaging Type, ND: Not Detected
